# Surgeon General Woodworth

**Published:** 1879-04

**Authors:** 


### Surgeon-General Woodworth.

The sudden death of the Supervising Surgeon-General of the Marine Hospital service will be received with deep sorrow by the Medical profession, as well as by the whole country. He has been the acceptable and efficient head of an important department of government, and his loss will be widely felt. His active efforts in regard to State Medicine during the past year have attracted the attention of the profession and public, and gained for him the respect and appreciation which he so richly deserved.

Doctor JOHN MAYNARD WOODWORTH was born at Big Flats, Chemung County, N. Y., August 15, 1837. He was taken to Illinois by his parents, and was educated at the University of Chicago. He first studied pharmacy, and attended the lectures on medicine and chemistry at Rush Medical College. He afterwards entered upon the regular study of medicine in that institution. The winters of 1859, 1860, and 1861 he spent in the Smithsonian Institution, working under the personal supervision of Prof. Spencer F. Baird. He graduated in 1862 at the Chicago Medical College. He immediately entered active service in the army of the Union as an assistant post surgeon at Camp Douglas, Illinois, and shortly after was appointed assistant surgeon of volunteers, and joined General Sherman in the field near Corinth, remaining with his command until the Union armies were mustered out in 1865. In 1863 he was promoted to the rank of surgeon, and assigned to duty as medical inspector of the fifteenth army corps, and afterward medical inspector and medical director of the army of the Tennessee. During Sherman's march to the sea he was brevetted lieutenant-colonel. 1865 he visited Europe, and in 1866 he established himself in Chicago. In 1871 he was appointed surgeon-general of the marine hospital of the United States, a position which he held up to the time of his death.

